# Could Dietary Black Soldier Fly Meal Inclusion Affect the Liver and Intestinal Histological Traits and the Oxidative Stress Biomarkers of Siberian Sturgeon (*Acipenser baerii*) Juveniles?

**DOI:** 10.3390/ani10010155

**Published:** 2020-01-16

**Authors:** Christian Caimi, Laura Gasco, Ilaria Biasato, Vanda Malfatto, Katia Varello, Marino Prearo, Paolo Pastorino, Maria Cristina Bona, Danila Raffaella Francese, Achille Schiavone, Antonia Concetta Elia, Ambrosius Josef Martin Dörr, Francesco Gai

**Affiliations:** 1 Department of Agricultural, Forest and Food Sciences, University of Torino, Largo P. Braccini 2, 10095 Grugliasco, Italy; christian.caimi@unito.it (C.C.); laura.gasco@unito.it (L.G.); vanda.malfatto@unito.it (V.M.); 2 Veterinary Medical Research Institute for Piedmont, Liguria and Aosta Valley, Via Bologna 148, 10154 Torino, Italy; katia.varello@izsto.it (K.V.); marino.prearo@izsto.it (M.P.); paolo.pastorino@izsto.it (P.P.); mariacristina.bona@izsto.it (M.C.B.); danilaraffaella.francese@izsto.it (D.R.F.); 3 Department of Veterinary Sciences, University of Torino, Largo P. Braccini 2, 10095 Grugliasco, Italy; achille.schiavone@unito.it; 4 Department of Chemistry, Biology and Biotechnology, University of Perugia, Via Elce di Sotto 8, 06123 Perugia, Italy; antonia.elia@unipg.it (A.C.E.); ajmartindoerr@libero.it (A.J.M.D.); 5 Institute of Sciences of Food Production, National Research Council, Largo P. Braccini 2, 10095 Grugliasco, Italy; francesco.gai@ispa.cnr.it

**Keywords:** gut histochemistry, *Hermetia illucens*, insect meal, liver histology, oxidative stress biomarkers, Siberian sturgeon

## Abstract

**Simple Summary:**

Insect meal is a suitable alternative to fishmeal (FM) in aquaculture feed. In recent years, numerous authors have studied the effects of insect meal as a substitute for fishmeal on fish growth performance, while only a few papers investigated its influence on the physiology and morphology of the digestive system and the oxidative status. The present study evaluated the effects of dietary highly defatted *Hermetia illucens* larva meal (H) inclusion and a vegetable protein based diet (VEG) on histological traits of liver and distal intestinal and oxidative stress biomarkers of liver and kidney in Siberian sturgeon juveniles. The results show that both the VEG and the H diets did not influence the liver and gut histology, but the highest inclusion level of H led to changes in oxidative stress biomarkers. Overall, these findings highlighted the possibility to include up to 18.5% of H as FM replacement in Siberian sturgeon diets without affecting the health status of fish.

**Abstract:**

The trial investigates if a highly defatted *Hermetia illucens* larva meal (H) at two dietary inclusion levels and a vegetable protein based diet (VEG) influences the normal gut and liver histology and the oxidative stress biomarkers in liver and kidney of Siberian sturgeon juveniles. Fish were fed four diets: one control diet (H0) containing 70% of fishmeal (FM), two diets including 18.5% (H185) and 37.5% (H375) of highly defatted H in substitution for 25% and 50% of FM, and one vegetable protein based diet (VEG). At the end of a growth trial, 12 fish per treatment were sacrificed by over-anaesthesia to collect 12 liver and 5 distal intestine samples for histological analyses, as well as 12 liver and kidney samples for biochemical analyses. The H and VEG diets did not significantly affect the histology of liver and distal intestine, but alterations of the oxidative stress biomarkers were detected at the highest inclusion level of H (37.5%). In order to avoid unfavorable effects on the fish health, an inclusion level up to 18.5% of H is recommended for Siberian sturgeon juveniles.

## 1. Introduction

The constant increasing rate of aquaculture production leads to a consequent increase in volume of fish feeds. Currently, aquaculture is the 4% of the global feed production accounting for about 44 million tons [1] with a constant global growth of about 4% in recent years. To ensure a proper growth of farmed fish, the use of high quality feed ingredients in order to guarantee the optimal feed utilization and constant productivity is necessary. Proteins are the most important macronutrients for animals, but represent the most expensive component in feed production [2]. For many years, fishmeal (FM) has been the primary protein source in feed for carnivorous fish, thanks to its high protein level, adequate aminoacid profile, high palatability, and digestibility [2]. 

In the last few years, the increasing price and low eco-sustainability of FM have prompted the focus towards new sustainable protein sources. The replacement of FM with vegetable proteins (VP) has been widely investigated [2]. The use of VP is advantageous due to their lower price and continuous availability, but despite this, the imbalanced amino acid profile, the high carbohydrate content, and the presence of antinutritional factors (ANFs) leads to a lower efficiency in feeds utilization, which makes these meals unsuitable for many species [2]. In some species, in particular carnivorous fish, it has been observed that the inclusion of VP as primary protein sources negatively affects growth performance [3,4] and can lead to intestinal dysfunction [5,6,7]. These effects have mainly been attributed to the presence of ANFs as protease inhibitors, phytates, saponins, and lectin able to decrease the nutrient utilization and induce an inflammatory response [2,8,9]. Additional FM substitutes have been identified in the terrestrial processed animal proteins (PAP), such as blood meal, poultry by-product meal, feather meal, and meat and bone meal. PAPs are characterized by an optimal amino acid profile and have shown a protein digestibility like that of FM [10]. Despite this, PAPs have a high variability due to the quality of raw materials and technological processes that limit their use in feeds production [11]. 

Starting from July 2017, the European Commission has approved the use of insect meals (IM) as protein sources in aquaculture [12]. Compared to FM and other alternative protein sources, the IM production has some advantages: insects can convert low value organic wastes into protein and fat [13], their production leads to lower emission of greenhouse gases, and they have a lower water footprint [14]. From a nutritional point of view, IM are rich in proteins, with a good essential amino acid (EAA) profile, and they have a good content of fat, vitamins, and minerals [15]. In the last few years, the potential of IM as partial or complete replacer of FM has been investigated [15] with interesting results both in freshwater fish such as rainbow trout (*Oncorhynchus mykiss*) [16,17], Siberian sturgeon (*Acipenser baerii*) [18,19], Jian carp (*Cyprinus carpio* var. Jian) [20], Yellow catfish (*Pelteobagrus fulvidraco*) [21], Nile tilapia (*Oreochromis niloticus*) [22,23] North African catfish (*Clarias gariepinus*) [24] and mandarine fish (*Siniperca scherzeri*) [25], and marine fish, as in European seabass (*Dicentrarchus labrax*) [26,27], gilthead sea bream (*Sparus aurata*) [28], Japanese seabass (*Lateolabrax japonicus*) [29], Atlantic salmon (*Salmo salar*) [30,31,32], and meagre (*Argyrosomus regius*) [33].

In addition to the evaluation of growth performance associated with a new feed ingredient, other aspects such as the physiology and morphology of the digestive system and the changes in oxidative status are also important parameters to investigate. Intestine is the main site of feed digestion and nutrient absorption, and its health status is capable of greatly affecting the utilization of dietary nutrients [16]. Therefore, a healthy digestive system is fundamental for the fish welfare and growth. Moreover, liver is one of the most important organs involved in nutrient metabolisms [34]. For the above-mentioned reasons, the assessment of the morphological alterations that may occur in these organs is crucial to guarantee the nutritional efficiency of the diet [16,35]. Another important aspect to consider is the role of the intestinal mucins, which are glycoproteins produced by the goblet cells (GC) of the gut mucosa. In particular, the neutral mucins promote the absorption and transportation of nutritive molecules in the plasma membrane [36], also performing an important role in the process of absorption of carbohydrates and fatty acids [37]. Histology together with other physiological indicators can be used to provide knowledge on the tolerance of fish species to new feed ingredients. 

Oxidative catabolism and nutritional factors may produce changes in levels of oxidative stress biomarkers [38]. Oxidative stress is generated by an imbalance between reactive oxygen species (ROS) and the antioxidant defenses of organism. Mechanisms that involve superoxide dismutase (SOD), catalase (CAT), and glutathione peroxidase (GPx) are important protective metabolic pathways and serve as biomarkers of oxidative stress. Both SOD and CAT enzymes catalyze the breakdown of ROS-generating O_2_^−^ and hydrogen peroxide (H_2_O_2_), respectively. The GPx enzyme reduces either H_2_O_2_ or organic peroxides, whereas glutathione reductase (GR) is involved in regeneration of reduced glutathione (GSH) from oxidized glutathione (GSSG). The metabolites produced from exogenous compounds metabolism by the superfamily of enzymes of cytochrome P450 (CYP) of phase I are conjugates with polar endogenous constituents in phase II producing water-soluble conjugates that are easily excreted. These reactions are catalyzed by glutathione S-transferase (GST) enzyme.

Nevertheless, it is already known that feeding behaviors, as well as environmental conditions may affect the oxidative homoeostasis in fish. Antioxidant defense in fish can vary changing from a marine-based to a plant-based diet; hence, lower transcriptional levels of SOD and reduced GSH concentration and CAT activity can be found in fish liver [39]. Although published data are available as far as the effects of vegetable oils or plant proteins on oxidative stress biomarkers of fish are concerned [39,40,41,42,43], there is still scanty information about the effects of insect inclusion [16,20,44,45,46].

This study aims firstly to investigate the effects of a dietary highly defatted *Hermetia illucens* larva meal (HIM) inclusion and a vegetable protein based diet (VEG) on histological traits of liver and distal intestinal sections of sturgeons and secondly to assess if changes in levels of biomarkers of oxidative stress (superoxide dismutase, catalase, glutathione peroxidases, glutathione reductase, ethoxyresorufin O-deethylase, glutathione S-transferase, and malondialdehyde, an indicator of lipid peroxidation) in liver and kidney are related to H and VEG diets.

## 2. Materials and Methods

A growth trial was conducted at the experimental facility of the Department of Agricultural, Forest and Food Sciences (DISAFA) of the University of Torino (Italy) as reported in Caimi et al. [19]. The experimental protocol was designed according to the guidelines of the current European Directive (2010/63/EU) [47] on the protection of animals used for scientific purposes and approved by the Ethical Committee of the University of Turin (Italy) (protocol N° 143811).

### 2.1. Experimental Diets and Fish Management

The experimental design, diets, and fish management are described in detail by Caimi et al. [19]. Briefly, four isonitrogenous (crude protein—CP: 50.5 g 100 g^−1^, as it), isolipidic (ether extract—EE: 12.4 g 100 g^−1^, as it), and isoenergetic (gross energy—GE: 20.84 MJ kg^−1^, as it) diets were formulated (Table 1) as follows: one control diet (HIM0) containing a high level (70%) of FM, two diets contained increasing inclusion levels of highly defatted HIM (Hermetia Deutschland GmbH & Co. KG, Baruth/Mark, Germany) in substitution for 25% (HIM25) and 50% (HIM50) of FM (corresponding to an inclusion level of 18.5% and 37.5%, as fed basis, respectively), and one vegetable protein based diet (VEG), formulated to mimic currently available commercial feeds (containing 32% of FM and 49% of plant protein sources).

To conduct the trial, a total of 440 fish were lightly anesthetised (MS222 100 mg L^−1^; PHARMAQ Ltd., Fordingbridge, UK), individually weighed, and randomly allocated to 16 tanks (22 fish per tank). Fish were fed in quadruplicate with the four experimental diets as detailed in Caimi et al. [19]. In the growth trial, an additional treatment where FM was completely substituted by including 75% of the insect meal (HIM100) was also formulated and distributed to the same number of tank replicates. However, due to a very low diet acceptance and in respect of the fish welfare, this treatment was stopped and excluded from any further investigations. To give a brief summary about the fish growth performance, sturgeons fed HIM50 showed a reduced feed consumption compared to HIM0 diet (2823.15 and 3003.04 g DM, respectively), thus resulting in a diminished final body weight (141.94 g and 159.32 g, respectively), weight gain (117.73 g and 135.12 g, respectively), and specific growth rate (1.48% and 1.59%/day, respectively).

At the end of the growth trial (118 days), fish were fasted for 24 h and three fish per tank (12 fish per treatment) were sacrificed by over-anaesthesia (MS-222-PHARMAQ Ltd., Fording bridge, UK; 300 mg L^−1^) to collect 12 portions of liver and 5 distal intestines (spiral valve) for histological analyses and 12 portions of liver and kidney for biochemical analyses.

### 2.2. Sampling and Histological Investigations

Sections of liver and distal intestine (spiral valve) were collected and fixed in 10% buffered formalin from 12 and 5 fish/treatment, respectively. The collected tissues were dehydrated in a graded ethanol series and embedded in paraffin wax blocks. Sections of 4 ± 2 μm thicknesses were cut with a microtome (Leica SM2000R, Leica Biosystem, Wetzlar, Germany), mounted on glass slides, and stained with Mayer Haematoxylin & Eosin (HE). The HE liver sections were examined by means of light microscopy in order to evaluate the following histological changes: nuclear displacement, cytoplasm vacuolization, lymphocytic infiltrates, and necrotic tissue areas. The observed histopathological findings on HE liver sections were assessed using a four-graded semi quantitative scoring system as follows (Table 2): absence of alterations (score 0); mild alterations (score 1); moderate alterations (score 2); severe alterations (score 3).

Gut sections were stained with the periodic acid-Schiff (PAS), which identified the neutral mucins in magenta [49]. One slide per intestinal section was examined by light microscopy. Five randomly selected high power fields per each slide were captured with a Nikon DS-Fi1 digital camera to a Nikon microscope (Nikon Instruments Europe B.V., Milan, Italy) using a 10× objective lens and NIS-Elements Ar 4.50.0064 Software (Nikon Instruments Europe B.V., Italy) was used for image capturing. The villus height (Vh) was evaluated on 5 villi per captured field (25 villi/sample) and the total number of PAS-positive goblet cells (GC) was counted on the five captured fields, according to Baeza-Arino et al. [35] and Pryor et al. [50]. Gut sections were also stained with Mayer Haematoxylin & Eosin (HE) and examined by means of light microscopy in order to evaluate the lymphocytic infiltrates using the above-mentioned four-graded semi quantitative scoring system (Table 2).

### 2.3. Biochemical Analyses

Oxidative stress biomarkers were evaluated in liver and kidney of each sample by spectrophotometer analysis (Varian Cary spectrophotometer at constant temperature of 25 °C) [16]. 

For malondialdehyde (MDA) analysis, sample was homogenized (1:7 p/v) in 20 mM Tris/HCl buffer pH 7.4, and 0.5 M butylated hydroxytoluene (BHT), centrifuged at 3000× *g* for 10 min at 4 °C. The supernatant was derivatized in 1-methyl-2-phenylindole (10.32 mM in acetonitrile/methanol diluted 4:1), concentrated HCl and dilution buffer (Tris/HCl, pH 7.4), sample or MDA standard (0–4 μM of 1,1,3,3-tetramethoxypropane). All samples were incubated for 60 min at 45 °C and then centrifuged at 15,000× *g* for 10 min, 4 °C. Concentration of MDA was read at 586 nm. Results were reported as nanomoles per gram of tissue. 

For all enzyme analysis, except for ethoxyresorufin O-deethylase (EROD), tissue was homogenized by an Ultra Turrax homogenizer in 100 mM potassium phosphate buffer (KP) pH 7.5 and 2.5% sodium chloride (NaCl), aprotinin 0.008 TIU/mL and 0.1 mg/mL bacitracin. Homogenates were centrifuged at 11,000× *g* for 45 min and then at 50,000× g for 90 min. 

Superoxide dismutase (SOD) activity was performed at 550 nm in 50 mM Na_2_CO_3_, pH 10, 0.1 mM EDTA, 500 mM cytochrome C and 1 mM hypoxantine and xantine oxidase. The reduction of cytochrome C by the xantine/hypoxantine system was measured versus a standard curve of SOD units or 50 µL of sample (diluted 1:50). One unit of SOD is defined as the amount of enzyme necessary to inhibit 50% of the reduction of cytochrome C.

Catalase (CAT) activity was performed at 240 nm (ε = −0.04 mM^−1^cm^−1^) following the decrease in absorbance of H_2_O_2_. The assay was carried out in a 100 mM NaH_2_PO_4_ + Na_2_HPO_4_ buffer pH 7 and 24 mM H_2_O_2_ and 10 µL of sample. 

Glutathione peroxidase (GPx) activity was measured at 340 nm (ε = −6.22 mM^−1^cm^−1^) in a 100 mM NaH_2_PO_4_ + Na_2_HPO_4_ buffer pH 7.5, 1 mM EDTA, 0.12 mM NADPH (β-Nicotinamide adenine dinucleotide), 2 mM GSH, 1 mM NaN_3_, 1 U of GR (glutathione reductase), and 0.6 mM H_2_O_2_ and 20 µL and 50 µL of sample for liver and kidney, respectively. The assay follows the oxidation of NADPH. 

Glutathione S-transferase (GST) activity was performed at 340 nm (ε = 9.6 mM^−1^cm^−1^) using CDNB (1-chloro-2,4-dinitrobenzene) as substrate. The assay was measured at 340 nm in 100 mM NaH_2_PO_4_ + Na_2_HPO_4_ buffer 100 mM pH 6.5, 2 mM GSH and 2 mM CDNB and 20 µL of sample. 

Glutathione reductase (GR) was measured at 340 nm (ε = −6.22 mM^−1^cm^−1^) in 100 mM NaH_2_PO_4_ + Na_2_HPO_4_ buffer 100 mM pH 7, GSSG (oxidized glutathione) 1 mM and NADPH 0.06 mM and 25 µL and 50 µL of sample for liver and kidney, respectively. The assay measured the decrease in absorbance due to the oxidation of NADPH.

The EROD activity was assessed on S9 cytosolic fraction after the homogenization in 100 mM KP, pH 7.5 with 0.15 M KCl and 1 mM EDTA (1:5). Enzyme activity was measured spectrofluorimetrically following resorufin production at 535/585 subsequent incubation in 100 mM KP buffer, pH 7.5, 4 mM 7-ethoxyresorufin, and 0.25 mM NADPH for 5 min and 100 µL of sample. The concentration of the protein in the cytosol was determined according to Lowry et al. [51] and was used to normalize the biomarker levels.

### 2.4. Statistical Analyses

Prior to statistical analysis, all the obtained data were evaluated for normality of distribution by Shapiro–Wilk’s test and when the assumption was not satisfied a logarithmic data transformation was applied. The Vh, number of GC and biomarker data were analysed by one-way ANOVA using IBM SPSS Statistics v. 25.0 for Windows. 

The following model was used: Y_ij_ = μ + D_i_ + ε_ij_, where Y_ij_ = observation; μ = overall mean; D_i_ = effect of diet (HIM0, HIM25, HIM50, VEG); ε_ij_ = residual error. 

Tukey’s HSD test was performed to discriminate the differences between the experimental treatments and the control groups. The histopathological scores were analysed by means of the Kruskal–Wallis test (post-hoc test: Dunn’s Multiple Comparison test). Results are given as mean and SD. The criterion for significance was set at *p* < 0.05.

## 3. Results

### 3.1. Histological Investigations

**Figure 1:**
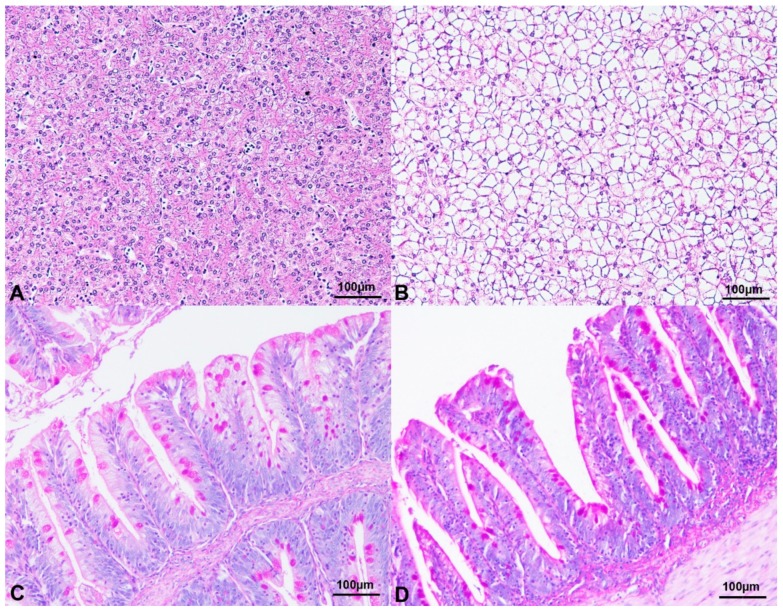
Histological and histochemical findings of Siberian sturgeon in the current trial. (**A**) Liver, Haematoxylin & Eosin stain, 10× magnification. HIM0 diet. Occasional, multifocal vacuolization of the hepatocytes. (**B**) Liver, Haematoxylin & Eosin stain, 10× magnification. Severe and diffuse vacuolization of the hepatocytes. VEG diet. (**C**) Spiral valve, 10× magnification. Numerous goblet cells strongly reacting to periodic acid-Schiff (PAS) stain are evident along the spiral valve epithelium. HIM25 diet. (**D**) Spiral valve, 10× magnification. Several PAS-positive goblet cells are identified in the spiral valve villi. VEG diet. Abbreviations: H, *Hermetia illucens* larva meal; VEG, vegetable protein based diet.

Dietary HIM inclusion did not significantly affect (*p* > 0.05) the liver histopathological changes (Table 3, Figure 1a,b). Analogously, the Vh and the number of GC showed no significant differences (*p* > 0.05) among the dietary treatments (Table 4, Figure 1c,d). The spiral valve also displayed no significant lymphocytic infiltrates in all the dietary treatments (Table 2).

### 3.2. Oxidative Stress Biomarkers

The effects of experimental diets on biomarkers of oxidative stress of Siberian sturgeon are shown in Table 5.

The MDA concentrations in liver and kidney of control and HIM and VEG fed diets did not noticeably change (*p* > 0.05). The two HIM diets produced a dose-dependent increase (50%) of SOD activity in mainly kidney (*p* = 0.001), whereas HIM50 caused a lower (30%) GPx activity in mainly liver (*p* = 0.027). GR activity increased (35%) with the highest HIM inclusion in liver (*p* = 0.001), while the same activity in kidney was higher (40%) at both HIM doses (*p* = 0.026). Higher EROD activity was found in kidney of HIM25 (6 fold, *p* < 0.001) and HIM50 (4 fold, *p* = 0.005), whereas no changes were found for the enzyme level in liver of HIM groups (*p* > 0.05). GST activity was higher (50%, *p* = 0.001) in kidney of HIM50 and did not change in liver of both HIM groups, compared to own controls (*p* > 0.05).

The levels of biochemical indicators in both tissues of sturgeons fed the VEG diet showed higher levels of CAT (40%) in both tissues and mainly in kidney (*p* = 0.003), of GPx (40%, *p* = 0.003) and GR (45%, *p* = 0.003) in kidney, and lower GST (35%, *p* = 0.006) activity in liver.

## 4. Discussion

### 4.1. Histological Investigations

One of the limiting factors of including insect meal in fish feed is their chitin content, which is able to determine gut and liver histological changes [16]. In particular, chitin could affect liver lipid accumulation [20] and may induce intestinal inflammation [52]. The histological effects on liver and gut by HIM utilization in diets for different fish species have already been characterized, with different results being obtained until now [16,20,24,31]. Our results showed that up to 37.5% of highly defatted HIM can be included in Siberian sturgeon feed without any negative effects on spiral valve and liver histology.

No histopathological alterations were observed in the liver of Siberian sturgeons fed HIM based diets. This result is in agreement with those observed in Japanese seabass [29], Atlantic salmon [30,31], zebrafish (*Danio rerio*) [52], and rainbow trout [16] fed with HIM meal. On the contrary, Li et al. [20] observed significant alterations in liver (decrease hepatopancreas fat deposition and increase in mild necrosis and apoptosis of hepatocytes) in Jian carp (*Ciprinus carpio* var. Jian) fed 7.9% inclusion level of HIM. Another recent study shows that inclusion level up to 40% of mopane worm meal (*Imbrasia belina*) worsened liver degradation, probably caused by the high fiber content in the mopane worm [24].

In most fish species, the dynamics of absorption decrease proceeding towards the posterior tract of the intestine. Since in sturgeon the maximum nutrient absorption takes place in the posterior intestine (spiral valve) [53], this segment was herein considered. In the present study, no significant differences were observed between the Vh and the number of GC between HIM0, VEG, and HIM-fed sturgeons. This is in agreement with what was reported in rainbow trout [16,54], post smolt Atlantic salmon [30], and Japanese seabass [29] fed with HIM meal. Another study conducted in Siberian sturgeon fed with HIM-based diet [18] showed that in the proximal intestine, the inclusion of 15% of full-fat HIM did not affect the villus height, but the authors reported a reduction in the thickness of mucosa and increase in muscle layer thickness—changes that have been associated with an enhancement of the digestion and absorption process. 

An increase in the number of GC in the distal intestine is usually associated with an immune reaction during the inflammation process and a decrease in nutrient absorption [35]. These gut mucin dynamics are frequently related to high inclusion levels of VP sources in fish diets [7,35]. In particular, the use of VP may cause morphological alterations capable of affecting the optimal nutrient absorption [6,55]. However, the effects of VP usually vary on varying the species, the protein source, and the level of inclusion [35,56,57,58]. Based on the authors’ knowledge, the effects of VP on the liver and gut histology of sturgeon have been only characterized by Kittel et al. [59]. In particular, the authors formulated four experimental diets for shovelnose sturgeon (*Scaphirhynchus platorhynchus*) containing increasing levels of soybean meal (from 0 to 51.23%) as partial replacement of FM and highlighted histopathological signs of distal enteritis in fish fed soybean meal-based diets confirming what was reported by previous studies [6,34,55].

### 4.2. Oxidative Stress Biomarkers

The dietary inclusion of HIM meal in substitution of 25% and 50% of FM and VEG diet did not significantly alter the histological traits of liver and distal intestine sections suggesting no adverse effect on digestive capacity of Siberian sturgeons. However, all the experimental diets (HIM25, HIM50 and VEG) have led to a disturbance of some oxidative stress indicators in liver and kidney of sturgeons.

Both kidney and liver tissues chosen for this study have high potential for ROS production and response of the oxidative stress biomarkers in fish is tissue-specific [60,61,62,63].

Each biomarker, under chemical or physical pressure, is prone to change and, within the different treatments, the response of all biochemical parameters can be dissimilar. In order to deepen the knowledge on the effects of HIM and VEG diets on sturgeons, a panel of physiological and biochemical markers was thus applied. Fish fed the HIM (25% and 50% substitution) and VEG diets showed increased levels of several biomarkers in liver and especially in kidney. Although HIM25 diet led to sporadic changes in renal enzymes activity, altered detoxifying response occurred more frequently in sturgeon fed HIM50. The GPx showed lowest value in both liver and kidney of specimens following HIM50 diets. However, no marked lipid peroxidation was observed through the experiment.

In the present study, the altered activity of several antioxidant enzymes, mainly in sturgeon fed HIM50, may be related to the diet’s composition. Hepatic increased levels of antioxidant enzymes, such as CAT, GPx, and GR, have been previously associated to protein- or lipid-rich FM diets in Adriatic sturgeon *Acipenser naccarii* [64]. Although HIM25 and HIM50 were isoproteic, isolipidic, and isoenergetic, differences in fatty acid profile were found compared to HIM0 diet [19]. Indeed, the levels of total fatty acids (TFA) were consistently higher in HIM50 compared to HIM0 (8025.84 vs. 6276.23 mg/100 g dry matter (DM), respectively), as well as saturated fatty acids (SFA—2794.26 vs. 1989.84 mg/100 g DM, respectively), and monoinsatured fatty acids (MFA—3580.15 vs. 2545.82 mg/100 g DM, respectively). Moreover, the ratio PUFA/SFA in HIM50 diet was found lower than control diet (0.59 vs. 0.87 mg/100 g DM, respectively). Sturgeon fed HIM50 also showed a reduced feed consumption compared to fish fed HIM0 (2823.15 and 3003.04 g DM, respectively), resulting in a diminished final body weight (141.94 g and 159.32 g, respectively), weight gain (117.73 g and 135.12 g, respectively), and specific growth rate (1.48% and 1.59%/day, respectively) [19]. Starvation was previously associated with a triggered antioxidant capacity in Siberian sturgeon, mainly increasing the activity of SOD [65]. Similarly, in our study a higher SOD activity was found in specimen fed HIM50. We may thus assume that the raised SOD and GR levels in liver and kidney of *A. baerii* may be related to the different lipid composition and lower HIM consumption.

In addition, the polysaccharide chitin, one of the centerpieces of insect exoskeleton and present at higher levels (up to 1.92 g/kg) in HIM substituted diets, deserves attention. Chitin has been recognized as an antioxidant molecule, also preventing deleterious effects in various diseases [66,67] and boosting the SOD activity in orange-spotted grouper (*Epinephelus coioides*) [68]. Chitin role is very complex and, as for other antioxidants, such as selenium [69,70,71], may act as pro-oxidant when the optimal threshold is encompassed. In the present study, significantly higher chitin doses (1.92 g/kg) were found in HIM50 diet and may be related to the lower GPx activity in both liver and kidney of sturgeon. This outcome is not surprising and was previously discussed in rainbow trout (*Oncorhynchus mykiss*) as associated with the ability of polysaccharide to bind selenium present in the diets, both in inorganic (selenite and selenite) and organic (selenomethionine and selenocysteine) form [16]. In particular, selenocysteine is essential for GPx functioning since it contains a selenocysteine residue in its structure and its unavailability following chitin bond may have reduced the enzyme activity.

In the present study, GST was affected by both the included diets (HIM25, HIM50) and VEG, although different responses were observed. Similarly to our previous results in rainbow trout [16], GST was enhanced only in kidney of sturgeon fed the higher substituted diet. This outcome and the concomitant increase of EROD suggest a strengthening of the detoxifying ability in groups fed HIM50 and an important role in the biotransformation of lipophilic compounds. Increase in GST activity has been previously reported in African catfish liver fed with cricket meal (*Grillus bimaculatus*) [46], while no changes were measured in carp fed with domestic fly larva meal [44]. On the contrary, the sturgeon fed VEG showed a decreased GST level. A previous study showed that β-conglycinin, one of the major allergenic proteins present in soybean [72], can decrease GST activity in intestine and enterocytes of carp, concomitant with an increased expression of the main antioxidant genes [73]. The dropped hepatic GST levels measured in our study may suggest a pointing scenario, since the reactive intermediates produced by the phase I enzyme (CYP) may not be adequately removed.

Nevertheless, the unchanged MDA levels in both liver and kidney in sturgeon fed with both the categories of substituted diets indicate preserved antioxidant efficiency. The lack of lipid peroxidation following vegetable meal is not surprising. Peng et al. [74] measured a reduction in lipid peroxidation in fish fed with soybean meal, probably correlated with an increased storage of hepatic α-tocopherol. Likewise, Wang et al. [29] found a lower concentration of MDA in Japanese seabass fed H, revealing that this diet may improve the antioxidant status of fish. On the contrary, Ji et al. [75] showed that the amino acid deficiencies in diets substituted with higher silkworm pupae doses caused lipid peroxidation and oxidative damage, also altering the intestinal microvilli and hepatocytes structure in juvenile Jian carp. This outcome suggests that lipid peroxidation may be related to fish species and/or protein source.

In conclusion, the inclusion of a highly defatted HIM and VP does not significantly affect the histology of liver and distal intestine of Siberian sturgeon. Nevertheless, as unfavorable effects on antioxidant response were reported at 37.5% of HIM inclusion, an inclusion level up to 18.5% is recommended for sturgeons.

Considering the high longevity of the sturgeons, further investigations are required to observe the long-time effects of insect and vegetable meals on gut and liver histology and the oxidative stress biomarkers of Siberian sturgeon.

## Figures and Tables

**Table 1:** Ingredients and proximate composition of *H. illucens* larva meal and experimental diets (modified from Caimi et al. [19]).

Ingredients (%, as Fed)	Experimental Diets				
	HIM	HIM0	HIM25	HIM50	VEG
Fish meal (Chile, super prime) ^a^		70	52.5	35	32
HI larva meal ^b^		0	18.5	37.5	0
Wheat meal		14	12	10	0
Corn gluten meal		0	0	0	15
Soybean protein concentrate		0	0	0	20
Soybean meal		0	0	0	14
Starch gelatinized, D500		8	8	8	8
Fish oil		6	7	7.5	9
Vitamin mixture ^c^		1	1	1	1
Mineral mixture ^d^		1	1	1	1
**Chemical Composition (% as Fed) ^e^**					
DM	94.94	96.41	96.39	96.29	97.37
CP	62.51	50.29	50.65	50.2	50.87
EE	4.03	12.68	12.62	12.1	12.81
Ash	8.2	13.15	11.71	10.24	9.91
Chitin ^f^	4.97	nd	0.72	1.92	nd
NFE ^g^	20.29	23.88	24.15	25.55	26.41
GE (MJ kg^−1^)	20.76	19.77	19.65	20.64	20.44

Note: HI, *Hermetia illucens*; HIM, *Hermetia illucens* larva meal; VEG, vegetable protein based diet; DM, dry matter; CP, crude protein; EE, ether extract; NFE, Nitrogen free extracts; GE, gross energy; nd, not detected. ^a^ Purchased from Corpesca S.A. (Santiago, Chile). Proximate composition (g 100 g^−1^, as fed basis): 88.7 DM; 63.8 CP; 8.4 EE; 14.9 ash. ^b^ Purchased from Hermetia Deutschland GmbH & Co. KG (Baruth/Mark, Germany). ^c^ Vitamin mixture (IU or mg kg^−1^): DL-α tocopherol acetate, 60 IU; sodium menadionebisulphate, 5 mg; retinyl acetate, 15,000 IU; DL-cholecalciferol, 3000 IU; thiamin, 15 mg; riboflavin, 30 mg; pyridoxine, 15 mg; B_12_, 0.05 mg; nicotinic acid, 175 mg; folic acid, 500 mg; inositol, 1000 mg; biotin, 2.5 mg; calcium panthotenate, 50 mg (purchased from Granda Zootecnica S.r.l., Cuneo, Italy). ^d^ Mineral mixture (g or mg kg^−1^): dicalcium phosphate, 500 g; calcium carbonate, 215 g; sodium salt 40, g; potassium chloride, 90 g; magnesium chloride, 124 g; magnesium carbonate, 124 g; iron sulphate, 20 g; zinc sulphate, 4 g; copper sulphate, 3 g; potassium iodide, 4 mg; cobalt sulphate, 20 mg; manganese sulphate, 3 g; sodium fluoride, 1 g (purchased from Granda Zootecnica S.r.l., Cuneo, Italy). ^e^ Values are reported as mean of duplicate analyses. ^f^ Estimated as ADF–ADFN [48]. ^g^ Calculated as 100 − (CP + EE + Ash + Chitin).

**Table 2:** Semi quantitative scoring system adopted for the assessment of the liver and the intestinal histological traits.

Alteration	Score 0	Score 1	Score 2	Score 3
Liver				
Vacuolization	Absent	Mild, focal to multifocal vacuolization of the hepatocytes (<25% of the cells is affected)	Moderate, multifocal to diffuse vacuolization of the hepatocytes (>25%, but <50% of the cells is affected)	Severe, multifocal to diffuse vacuolization of the hepatocytes (>50% of the cells is affected)
Nuclear displacement	Absent	The nucleus of the hepatocytes is mildly displaced towards the cell membrane	The nucleus of the hepatocytes is moderately displaced towards the cell membrane	The nucleus of the hepatocytes is severely displaced towards (and adhered to) the cell membrane
Lymphocytic infiltrates	Absent	Mild, focal to multifocal interstitial and/or perivascular lymphocytic infiltrates (<25% of the liver parenchyma is affected)	Moderate, multifocal to diffuse interstitial and/or perivascular lymphocytic infiltrates (>25%, but <50% of the liver parenchyma is affected)	Severe, multifocal to diffuse interstitial and/or perivascular lymphocytic infiltrates (>50% of the liver parenchyma is affected)
Necrotic tissue area	Absent	Small, focal to multifocal areas of hepatocyte necrosis	Small to large, multifocal areas of hepatocyte necrosis	Large, multifocal to diffuse areas of hepatocyte necrosis
Spiral valve				
Lymphocytic infiltrates	Absent	Mild, focal to multifocal mucosal, submucosal and/or muscular lymphocytic infiltrates (<25% of the intestinal wall is affected)	Moderate, multifocal to diffuse mucosal, submucosal and/or muscular lymphocytic infiltrates (>25%, but <50% of the intestinal wall is affected)	Severe, multifocal to diffuse mucosal, submucosal and/or muscular lymphocytic infiltrates (>50% of the intestinal wall is affected)

**Table 3:** Effects of dietary *H. illucens* larva meal inclusion on liver histopathological findings of the sturgeons (*n* = 12).

Items	Experimental Diets				SEM	*p*-Value
	HIM0	HIM25	HIM50	VEG		
Liver						
Vacuolization	2.67	2.58	2.83	2.75	0.073	0.663
Nuclear displacement	1.50	1.83	1.92	2.08	0.100	0.215
Lymphocytic infiltrates	1.42	1.33	1.33	1.67	0.098	0.601
Necrotic tissue area	Absent	Absent	Absent	Absent		
Spiral valve						
Lymphocytic infiltrates	Absent	Absent	Absent	Absent		

Note: HIM, *Hermetia illucens* larva meal; VEG, vegetable protein based diet; SEM, standard error of the mean; *p*, probability. Data are expressed as mean of the histopathological scores.

**Table 4:** Effects of dietary *H. illucens* larva meal inclusion on gut morphology and neutral mucin-producing goblet cells of the sturgeons (*n* = 5).

Items	Experimental Diets				SEM	*p*-Value
	HIM0	HIM25	HIM50	VEG		
Vh (µm)	330.93	313.16	338.04	314.54	4.29	0.105
GC number	61.28	77.08	68.76	67.44	2.909	0.293

Note: HIM, *Hermetia illucens* larva meal; VEG, vegetable protein based diet; Vh, villus height; GC, Goblet cell; SEM, standard error of the mean; *p*, probability. Data are expressed as mean.

**Table 5:** Effects of dietary *H. illucens* larva meal inclusion and vegetable protein based diet on oxidative stress biomarkers in liver and kidney of sturgeons.

Items	Experimental Diets				SEM	*p*-Value
	HIM0	HIM25	HIM50	VEG		
Liver						
MDA	150.73	140.81	159.64	129.13	4.53	0.094
SOD	23.53 ^b^	20.18 ^b^	33.22 ^a^	26.84 ^a,b^	1.25	0.001
CAT	614.13 ^b^	644.23 ^b^	734.23 ^a,b^	836.06 ^a^	20.51	0.001
GPx	69.75 ^a^	55.71 ^a,b^	50.53 ^b^	57.16 ^a,b^	2.48	0.037
GR	30.07 ^b^	30.42 ^b^	46.63 ^a^	34.35 ^b^	1.59	0.001
EROD	13.22	17.96	10.6	15.01	1.06	0.077
GST	246.50 ^a^	239.30 ^a^	274.39 ^a^	155.80 ^b^	11.16	0.001
Kidney						
MDA	108.49	111.55	108.75	121.64	5.47	0.816
SOD	12.61 ^b^	18.51 ^a,b^	22.95 ^a^	18.82 ^a,b^	1.02	0.001
CAT	85.31 ^b^	81.10 ^b^	109.18 ^a,b^	134.15 ^a^	5.59	0.003
GPx	53.55 ^b,c^	67.78 ^b^	36.24 ^c^	83.64 ^a^	4.18	0.001
GR	16.64 ^b^	26.63 ^a^	25.94 ^a^	29.52 ^a^	1.36	0.003
EROD	6.34 ^c^	38.07 ^a^	22.42 ^b^	12.32 ^b,c^	3.49	0.001
GST	48.05 ^b^	62.55 ^b^	95.22 ^a^	52.60 ^b^	4.86	0.001

Abbreviations: HIM, *Hermetia illucens* larva meal; VEG, vegetable protein based diet; Data are reported as mean; SEM, standard error of the mean (*n* = 12). *p*, probability. Different superscript letters (^a,b^ for liver; ^a,b,c^ for kidney) indicate statistically significant differences (Tukey’s multiple comparisons test) between the experimental groups (HIM or VEG). MDA (nmol/g tissue); SOD (U/mg prot), CAT (µmol/min/mg prot), GPx, GR and GST (nmol/min/mg prot), EROD (pmol/min/mg prot).
